# A Novel Phenotype of the Factor 5 Gene Mutation (Homozygote Met1736Val and Heterozygote Asp68His) Is Associated With Moderate Factor V Deficiency

**DOI:** 10.3389/fmed.2022.870269

**Published:** 2022-06-09

**Authors:** Yueh-Shih Chang, Yi-Cheng Lan, Ya-Jyun Chen, Jen-Seng Huang, Chia-Ning Yang, Chi-Ying F. Huang, Kun-Yun Yeh

**Affiliations:** ^1^Division of Hemato-Oncology, Department of Internal Medicine, College of Medicine, Chang Gung Memorial Hospital, Keelung & Chang Gung University, Keelung, Taiwan; ^2^Institute of Clinical Medicine, National Yang Ming Chiao Tung University, Hsinchu, Taiwan; ^3^Institute of Precision Medicine, National Sun Yat-sen University, Kaohsiung, Taiwan; ^4^Institute of Biopharmaceutical Sciences, National Yang Ming Chiao Tung University, Hsinchu, Taiwan

**Keywords:** factor V deficiency, factor V gene, polymorphism, coagulopathy, Asia

## Abstract

**Background:**

Factor V (FV) deficiency is a rare disease, with a low incidence rate in Asia. Therefore, the *F5* mutation in the Taiwanese population is poorly understood.

**Methods:**

A Chinese family with FV deficiency was included, and the patient and his family members underwent mutation analysis. Then, patients from Keelung City (Taiwan) were screened for *F5* polymorphism; the Chang Gung Human Database was used to determine single-nucleotide variants in the non-FV-deficient patient population.

**Results:**

Eight mutation sites on the *F5* gene locus, including exon 16 homozygote Met1736Val and seven heterozygous mutations, including Asp68His, were found. Moreover, Met1736Val was found to be the dominant mutation in people living in the Taiwan community, and this result was compared with the records of the Chang Gung Human Database. The above-mentioned polymorphisms may result in a variable incidence of FV deficiency in Keelung City, thereby facilitating carrier diagnosis and prenatal diagnosis in most FV-deficient families.

**Conclusion:**

The homozygote Met1736Val and the co-inheritance of the Asp68His *F5* gene are unique and worthy of screening in FV-deficient patients.

## Background

Factor V (FV) deficiency, or parahemophilia, first described by Owren in 1947 (1), is an autosomal recessive bleeding disorder characterized by low FV coagulant activity and antigen levels. Factor V deficiency is a rare bleeding disorder, with an estimated prevalence of one per one million (2, 3). The phenotypic expression of FV deficiency is variable, with mutations showing different severities of bleeding symptoms. Both homozygous and heterozygous mutations may affect the severity of bleeding symptoms (4). Thus, identifying the gene polymorphism of *F5* will provide additional insights into the mechanisms underlying this variable clinical expression.

Human coagulation FV (proaccelerin or labile factor) plays an important role in mediating coagulation (5). Factor V is a single-chain, high-molecular-weight glycoprotein (330 kDa) primarily synthesized by hepatocytes and megakaryocytes. Plasma FV is mainly represented by precursor molecules, and 20% of it is stored in platelet alpha-granules (6). The domain architecture of *F5* is ordered as A1–A2–B–A3–C1–C2 (7). The human *F5* gene has been mapped to chromosome 1q23 and spans >80 kb (8), and the entire gene contains 25 exons and 24 introns. The messenger RNA encodes a leader peptide of 28 amino acids and a mature protein comprising 2,196 amino acids (9). The heavy chain (A1–A2) is encoded by exons 1–12, whereas the light chain (A3–C1–C2) is encoded by exons 14–25. The other part—the entire B domain—is encoded by exon 13. After proteolytic cleavage by thrombin or activated factor X (FXa), the B domain is removed, and FV is converted to its active form (FVa) (10, 11). One heavy chain and one light chain, linked together in the presence of Ca^2+^ ions, constitute the fully activated FV (12). Factor V, converted to its active form, binds to FXa in the prothrombinase complex that converts prothrombin to thrombin in the presence of calcium and a phospholipid membrane. Activated protein C (APC) inactivates FVa by cleaving the active factor in three additional arginine residues and requires FV in APC-mediated inactivation of factor VIIIa (13, 14). Thus, FV plays an important role in both pro-coagulant and protein C anticoagulant pathways (15). The dual role of FV is reflected in disorders associated with molecular defects in FV, which can be either hemorrhagic or thrombotic.

In 2018, the updated Human Genome Mutations Database (http://www.hgmd.org) displayed that the most homozygous or heterozygous mutations occur in the heavy (A1–A2) and light (A3–C1–C2) chains (~80% mutation annotated in these two chains). Another mutation profile of FV deficiency was reported by Paraboschi et al. in 2020 (16). In Taiwan, studies about FV deficiency are limited and focus on the A1 domain mutations, such as Asp68His and His147Arg (17). In this study, we report the cases of two patients with FV deficiency wherein we sequenced their *F5* genes. To facilitate a better understanding of FV deficiency in Taiwan, we traced their family history and analyzed the non-FV-deficient population.

## Methods

### Measurement of Prothrombin Time, Activated Partial Thromboplastin Time, Prothrombin Time, and FV Coagulant

Tests of prothrombin time (PT) and activated partial thromboplastin time (APTT) were performed in our laboratories using the Clauss method (18). The normal ranges for coagulation tests were determined according to the threshold used by the manufacturers of the reagents.

The FV activity in plasma was examined *via* a functional assay based on PT using human recombinant tissue factor (Instrumentation Laboratory Company, Bedford, MA). Plasma FV antigen was measured *via* a sandwich enzyme immunoassay (EIA; Hyphen Biomed, Andresy, France) using a sheep anti-human FV polyclonal antibody (6). The FV activity was expressed as a percentage of normal pooled plasma.

### Factor FV Exon Analysis

Before the gene analysis, we provided adequate information to the patient and his family regarding the reasons for the analysis. The study was reviewed and approved by our institutional review board (Nos. 200903793B0, 201102005A3D001, 201102005A3C102, and 201102005A3C103). After explaining the study details, all participants provided informed consent. We conducted a mutation analysis of the patient and his family members. To better understand the polymorphism of the *F5* gene, including its promoter, a total of 111 non-FV-deficient people were enrolled at the Keelung Chang Gung Memorial Hospital. The Ethylenediamine tetraacetic acid (EDTA)-anticoagulated blood samples were collected from the patient and his family members. Genomic DNA was isolated from whole blood using the QIAamp DNA Blood Mini kit (Qiagen, Hiden, Germany). All exons and exon–intron boundaries of the FV gene were amplified *via* the polymerase chain reaction (PCR) using a 50-μl reaction volume containing 0.5–1.0 μg DNA, 20 pmol/L of each primer, 0.2 mmol/L of each dNTP, 2.0 mmol/L MgCl_2_, and 1.5 U Taq DNA polymerase in an appropriate buffer. The polymerase chain reaction was performed in 30 cycles of 30 s at 94°C, 40 s at 50–60°C, and 40 s at 72°C, followed by an elongated extension of 7 min at 72°C. The PCR products were analyzed *via* direct DNA sequencing using the ABI PRISM 3700 DNA Sequencer (Applied Biosystems, Foster City, CA, USA). Table 1 shows the gene analysis results of the family. We also performed an *F5* polymorphism genotype analysis, including its promoter, and the results for the non-FV-deficient population are shown in Table 2 (25 exons of *F5*)/Supplementary Table 1 (*F5* promoter).

**Table 1:** The pattern of mutation and clinical symptoms in FV deficiency family.

	**Genotype**	**Patient 1**	**Patient 2**	**Father**	**Mother**
Exon 3 Asp68His	Heterozygote **g**ac → **c**ac	Heterozygote	Heterozygote	Heterozygote	
Exon 8 Met385Thr	Heterozygote a**t**g → a**c**g	Heterozygote	Heterozygote		Heterozygote
Exon13 Asn789Thr	Heterozygote a**a**c → a**c**c	Heterozygote	Heterozygote		Heterozygote
Exon13 Lys830Arg	Heterozygote a**a**a → a**g**a	Heterozygote	Heterozygote	Heterozygote	
Exon13 His837Arg	Heterozygote c**a**t → c**g**t	Heterozygote	Heterozygote	Heterozygote	
Exon13 Lys897Glu	Heterozygote **a**ag → **g**ag	Heterozygote	Heterozygote	Heterozygote	
Exon16 Met1736Val	Homozygote **a**tg → **g**tg	Homozygote	Homozygote	Heterozygote	Heterozygote
Exon25 Asp2194Gly	Heterozygote g**a**t → g**g**t	heterozygote	Heterozygote		
Clinical presentation
Age		19	20	51	44
Clinical symptoms		Easy bruising	1. Easy bruising 2. Postpartum hemorrhage	No bleeding episode	No bleeding episode
Factor V activity, % (50–150%)		3.2%	2%	58%	54%
PT: sec (range: 8–12)		22.2	13.7	10.2	10.4
APTT: sec (range: 25.0–31.3)		53.2	33.8	30.5	31.0

*Patient 1 and 2 both had the same clinical symptoms and the Factor V activity*.

*PT, Prothrombin time; APTT, Activated partial thromboplastin time*.

**Table 2:** Factor V polymorphism distribution for Keelung non-FV deficiency population, including 25 exons.

**Exon**	**Mutation**	**Heterozygote number**	**Homozygote number**
exon 3	Asp68His (D68H)	1/111 (0.9%)	0
exon 8	Met385Thr (M385T)	2/111 (1.8%)	0
exon 13	Asn789Thr (N789T)	0	0
	Lys830Arg (K830R)	27/111 (24.3%)	4/111 (3.6%)
	His837Arg (H837R)	27/111 (24.3%)	4/111 (3.6%)
	Lys897Glu (K897E)	27/111 (24.3%)	4/111 (3.6%)
exon 16	Met1736Val (M1736V)	47/111 (42.3%)	8/111 (7.2%)
exon 25	Asp2194Gly (D2194G)	4/111 (3.6%)	0

### Database Analysis of FV Polymorphism

We used the Chang Gung Human Database to determine single-nucleotide variants (SNVs) on exons 3, 8, 13, 16, and 25 in a non-FV-deficient patient population (including 391 individuals). Whole blood samples were collected from 391 patients from 2008 to 2018. All detected variants were filtered against a panel of 300 germline DNA samples (Chang Gung Human Database, an unpublished, institutional whole-genome database of normal controls). Table 3 lists non-synonymous and synonymous mutations. We used the database described in the previous studies (16, 19).

**Table 3:** The nonsynonymus mutations in non-FV deficiency patient population.

**Exon**	**refGene**	**AAChange**	**Frequency SNP**	
exon25	T6673C	X2225Q	0.00128	Stoploss
exon25	A6665G	D2222G	0.02046	Nonsynonymoussnv
exon24	A6452G	K2151R	0.00128	Nonsynonymoussnv
exon23	C6298T	R2100C	0.00128	Nonsynonymoussnv
exon23	G6277A	G2093R	0.00128	Nonsynonymoussnv
exon20	G5828C	G1943A	0.00128	Nonsynonymoussnv
exon17	G5558T	G1853V	0.01918	Nonsynonymoussnv
exon17	A5552C	E1851A	0.00128	Nonsynonymoussnv
exon17	A5431T	M1811L	0.00128	Nonsynonymoussnv
exon16	G5378T	R1793I	0.00128	Nonsynonymoussnv
exon16	A5290G	M1764V	0.23402	Nonsynonymoussnv
exon16	G5275C	D1759H	0.00128	Nonsynonymoussnv
exon13	C4210T	P1404S	0.12276	Nonsynonymoussnv
exon13	C4189T	L1397F	0.67008	Nonsynonymoussnv
exon13	T4000C	F1334L	0.00128	Nonsynonymoussnv
exon13	A3980G	H1327R	0.02174	Nonsynonymoussnv
exon13	C3853A	L1285I	0.09335	Nonsynonymoussnv
exon13	C3446T	S1149F	0.00384	Nonsynonymoussnv
exon13	A2998G	K1000E	0.00128	Nonsynonymoussnv
exon13	T2911C	W971R	0.00128	Nonsynonymoussnv
exon13	A2773G	K925E	0.21228	Nonsynonymoussnv
exon13	A2594G	H865R	0.21228	Nonsynonymoussnv
exon13	A2573G	K858R	0.21228	Nonsynonymoussnv
exon13	A2450C	N817T	0.02174	Nonsynonymoussnv
exon13	G2219A	R740Q	0.00128	Nonsynonymoussnv
exon13	A2032G	K678E	0.00384	Nonsynonymoussnv
exon10	A1601G	Q534R	1	Nonsynonymoussnv
exon10	G1538A	R513K	0.66624	Nonsynonymoussnv
exon11	T1699C	C567R	0.00128	Nonsynonymoussnv
exon8	T1238C	M413T	0.02558	Nonsynonymoussnv
exon7	C1106T	A369V	0.00512	Nonsynonymoussnv
exon7	A1000G	R334G	0.00256	Nonsynonymoussnv
exon6	C801A	F267L	0.00128	Nonsynonymoussnv
exon4	T398C	F133S	0.00128	Nonsynonymoussnv

*Among these nonsynoymous mutations, 8 of them marked in red have high (exon16: M1764V exon13: P1404S, L1397F, K925E, H865R, K858R; exon10:Q534R, R513K) in whole-exome sequencing of OSCC tumor-normal (fresh-frozen tumors and matched whole blood) database in Taiwan*.

### Factor V Protein Conformational Changes at a Specific Site of Met1736

We searched the RCSB Protein Data Bank (RCSB PDB: Homepage). We then used the BIOVIA Discovery Studio Visualizer version (BIOVIA, San Diego, CA, USA) to illustrate the mutation site and protein folding process.

## Results

### Clinical Course of FV-Deficiency Cases

The first patient was a 19-year-old man admitted to Chang Gung Memorial Hospital (Keelung branch) for an inguinal hernia operation. He had no history of spontaneous bleeding. Routine clotting tests confirmed a moderate FV deficiency. He received fresh frozen plasma before surgery, and no bleeding complications occurred.

**Figure 1:**
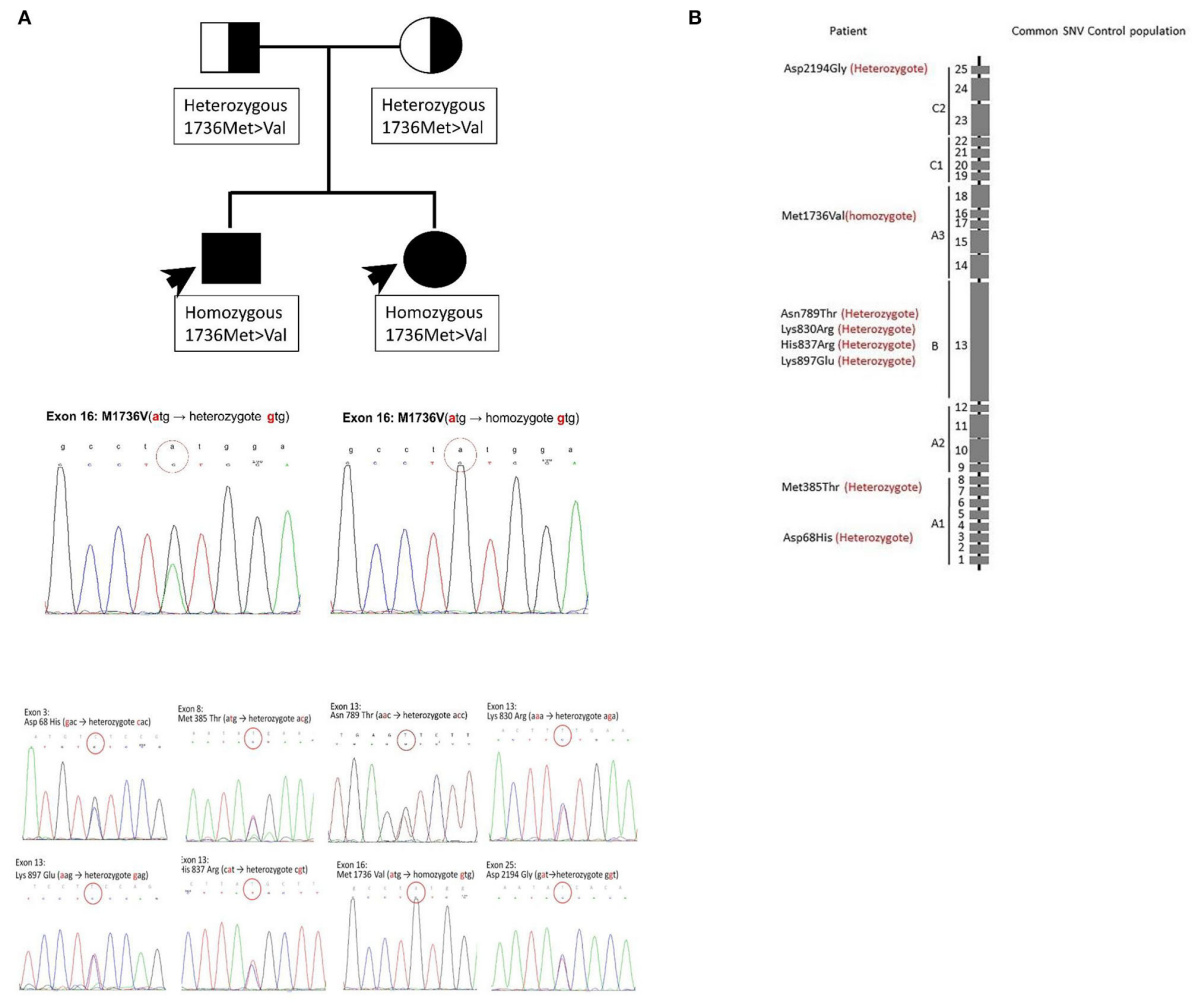
Mutation results of the patient and his family. In this family, two members had a coagulopathy disorder. **(A)** The pedigree chart. The FV deficiency was only noted in their children. The results of the *F5* gene analysis for their children are the same and shown at the bottom. Crucial mutations in this family. Homozygous Met1736Val and other seven heterozygous mutations coexisted. **(B)** Those mutation sites are demonstrated. The graph shows the absolute location of the mutation sites on the FV gene. The location of eight mutation sites on the chromosome. The mutation sites are demonstrated sequentially.

Before we commenced our study, we suggested that his family members be screened for coagulopathy and informed them about the impact of genetic mutation and the hereditary risk. His parents did not suffer from bleeding problems and denied consanguinity. The PT, APTT, and FV activity are shown in Table 1. Figure 1A shows the pedigree chart. The patient's 20-year-old sister mentioned that she was easily bruised but did not pay much attention to this problem. Compared with other coagulation factor deficiencies, bleeding due to FV deficiency is typically harmless. However, she had experienced a massive postpartum hemorrhage in 2017 at a gynecology clinic. The bleeding could not be stopped by emergency surgical intervention after she was transferred to our hospital. She received a fresh frozen plasma transfusion after the operation, which reduced the bleeding. After this episode, we informed them about the importance of prophylactic blood transfusion before undergoing any future surgical interventions.

### Detection of FV Deficiency Within a Family and Phenotype Identification

Both patients had a prolonged APTT and PT; the thrombin time was within the normal range. The plasma coagulation factor levels were normal, except for FV. The FV activity of patient 1 was only 3.2% (normal range, 50–150%), which indicated that this patient had moderate FV deficiency, and that of his sister was 2% (normal range, 50–150%). Although patient 1 denied any prior spontaneous bleeding episode, patient 2 suffered from easy ecchymosis. Their parents did not suffer from bleeding problems and denied consanguinity. Table 1 summarizes mutation patterns and clinical symptoms in the family.

### Gene Mutation Analysis of This Family

Only a limited number of mutations have been documented because of the large size and complexity of the coagulation *F5* gene (1q23) and its low prevalence. Almost all mutations identified to date have been unique and specific to the family. All 25 exons were assessed separately for each patient. We detected eight mutation sites in this study. Here, we report the combination of mutation sites in one patient and predict FV deficiency in his sibling carrying the same mutations.

First, we recorded eight mutation sites in the patient as follows: exon 3 (Asp68His), exon 8 (Met385Thr), exon 13 (Asn789Thr), exon 13 (Lys830Arg), exon 13 (His837Arg), exon 13 (Lys897Glu), exon 16 (Met1736Val), and exon 25 (Asp2194Gly) (Table 1). Only exon 16 expressed the homozygous genotype. Half of the mutations are reportedly heterozygous mutations on the B domain (exon 13). Frequent B domain mutations have been reported before, but their functions remain unclear (20, 21). Other gene mutations in exons 3, 8, and 25 were heterozygous. The pattern and location of each gene mutation are depicted in Figures 1A,B, respectively.

We then analyzed the *F5* gene in other family members and demonstrated its mutation pattern throughout the entire family (Table 1). Interestingly, the patient's sister had the same phenotype of the *F5* gene, i.e., exon 16 (Met1736Val), which was homozygous. Based on the FV level, the patient's sister also had moderate FV deficiency. A co-inheritance of Met1736Val FV gene mutation and seven polymorphisms were identified within this family. This study is the first to report many mutations detected in exon 13.

### Polymorphism Analysis of the Non-FV-Deficient Population

In the polymorphism screening, 111 non-FV-deficient participants were included. We focused on the eight mutation sites detected in our patients. The frequency of *F5* polymorphism is shown in Table 2. The frequency of heterozygote mutations ranged from 0.9 to 42.3%. The presence of a homozygous mutation was less frequent and only ranged from 0 to 7.2%. Among the eight mutation sites, the mutation of Met1736Val in exon 16 is more common than other mutation sites among people from Keelung City (up to 42.3%). Other hot mutation sites located on exon 13 include Lys830Arg, His837Arg, and Lys897Glu (24.3%). Less frequent mutation sites are Asp68His in exon 3 (0.9%), Met385Thr in exon 8 (1.8%), and Asp2194Gly in exon 25 (3.6%). Thus, we need more information on the prevalence of FV deficiency and polymorphism based on data available from other databases. To better understand the role of promoters, we performed an *F5* promoter gene analysis, the results of which have been provided in Supplementary Table 1. Seven promoter mutations were identified. The promoter bases that changed at −1,559 (–g), −1,506 (t → c), −1,487 (t → c), −790 (–c), and −95 (t → c) were considered to be polymorphic, given their high frequency in the Keelung non-FV-deficiency population. The frequencies of the other two mutations, −319 (a → c) and −281 (g → a), were 1 and 66.7%, respectively.

The SNVs of FV in the healthy Taiwanese population have not been analyzed previously. According to the eight mutation sites in the five exons, the aim was to determine the SNVs of the non-FV-deficient population to further elucidate the existence of mutations we found in the normal population. The results are shown in Table 3. By analyzing the sequence at the *F5* locus, we found 55 SNVs. The frequency of the SNVs can be found ranging from 1 to 66%. None of the SNVs were observed in the patient. Of the SNVs, 66% were non-synonymous amino acid substitutions. Some non-synonymous SNVs (Arg513Lys/A2, Pro1404Ser/B, Leu1397Phe/B, Lys925Glu/B, His865Arg/B, Lys858Arg/B, and Met1764Val/A3) were highly conserved in the Taiwanese population.

### Location and Influence of Met1736 on the FV Protein

**Figure 2:**
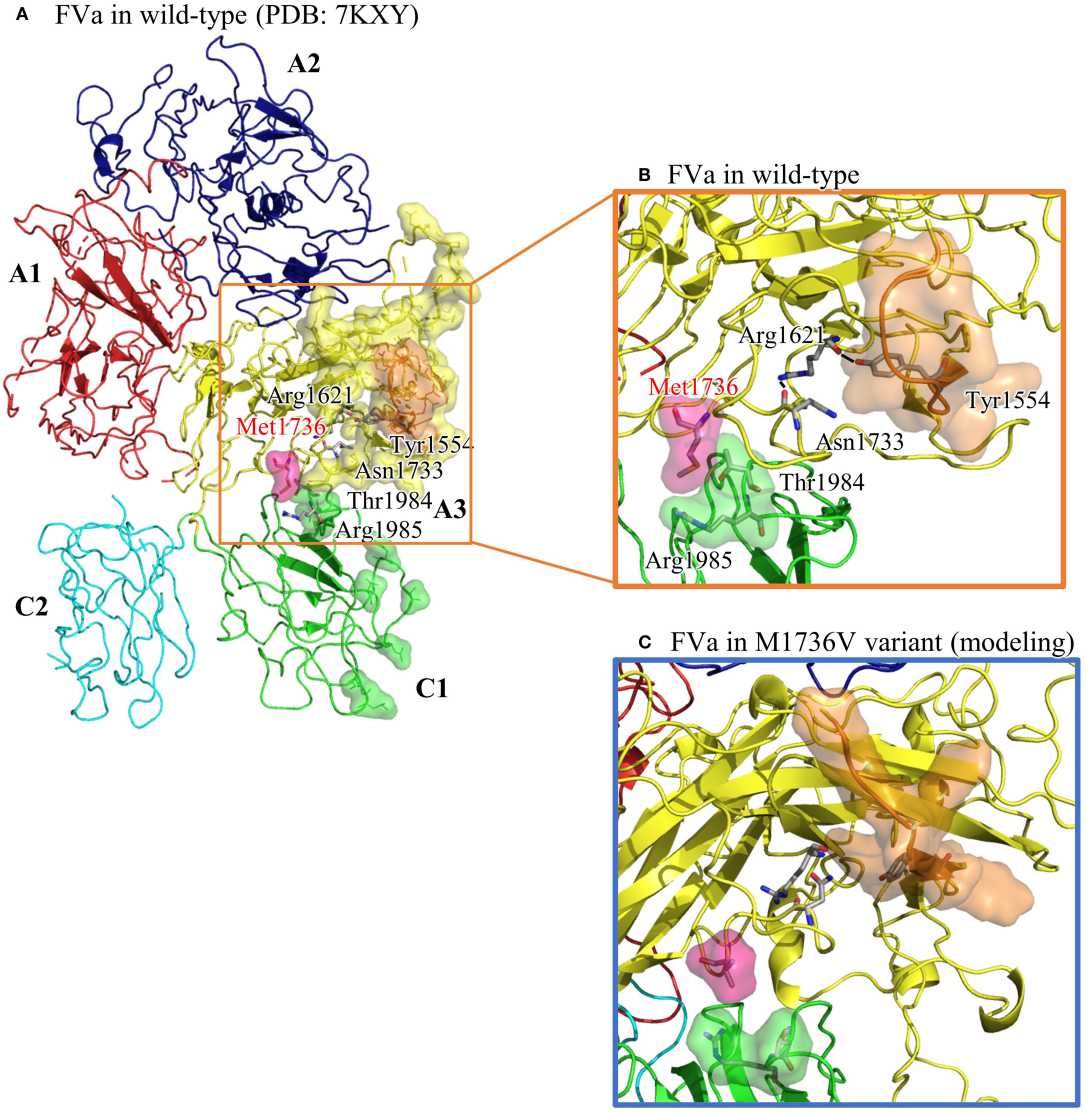
The structural analysis of FVa in wild type (in PDB: 7KXY) and in Met1736Val variant obtained by molecular modeling using GaMD. **(A)** The overall FVa structure is composed of A1 (in red), A2 (in navy blue), A3 (in yellow), C1 (in green), and C2 (in turquoise) domains. A3 residues interacting with FXa and prothrombin are encompassed by yellow surface, whereas C1 residues interacting with prothrombin are encompassed by green surface. The Asn1547–Asn1555 (N1547–Y1555) segment in A3 domain is highlighted in orange surface. **(B)** A close-up view of the wild type showing Met136 embedded by Thr1984 and Arg1985, and a well-maintained hydrogen bond network linking Asn1733, Arg1621, and Tyr1554. **(C)** A close-up view of the Met1736Val variant. Val1736 averts from Thr1984 and Arg1985, and Tyr1554 is no longer restrained by Arg1621, which possibly causes reorientation of the Asn1547– Tyr1555 segment.

The structural analysis of FVa in Protein Data Bank code, 7KXY, as shown in Figure 2A, suggests that the solvent-front Met1736 (on A3 domain) plays a dual role in (i) anchoring its nearby Thr1984 and Arg1985 (on C1 domain) and (ii) positioning the adjacent Asn1733 into the core of the A3 domain to secure a hydrogen bond network linking Asn1733, Arg1621, and Tyr1554. In a molecular modeling-based prediction by Schreuder et al., FVa's A3 and C1 domains are associated with FXa and prothrombin (22). In Figure 2B, we also highlight A3's residues interacting with FXa and prothrombin (encompassed by yellow and orange surface) and C1's residues interacting with prothrombin (encompassed by green surface and covering Thr1984). Notably, that part of the A3–FXa interface is made of the Asn1547–Tyr1555 segment (highlighted in orange surface), where most residues face outward to reach FXa. Rather than facing out, the aromatic ring moiety of Tyr1554 is embedded in the A3 domain core, and the hydroxy group forms a hydrogen bond with Arg1621, structurally stabilizing the Asn1547–Tyr1555 segment. In addition, T1984 is located on the FVa C3 domain and in the FVa–prothrombin interface. The interdomain interactions among Thr1984, Thr1985, and Met1736 shall help orient Thr1984.

To understand the structural impact due to the Met1726Val mutation, Gaussian accelerated molecular dynamics (GaMD)-enhanced sampling simulations were applied to the FVa structure carrying Val1736. The aforementioned wild-type FVa structure was used to create the Met1726Val variant by manually modifying Met1726 into a valine residue. With a previously used protocol (23), we collected a 100-ns long trajectory, as summarized in Figure 2C. Then Val1726 is detached from Thr1984 and Arg1985, resulting in a rearrangement of Thr1984 and Asn1986. The Arg1621–Tyr1554 linkage is lost and the Asn1547–Asn1555 segment moves to a new position. To summarize, the mutated Val1726 reorients the structural features essential for FVa–FXa–prothrombin complex formation.

## Discussion

A Taiwanese family with FV deficiency was included, and a novel homozygous mutation of Met1736Val in exon16 was found. Although symptom severity does not correlate with the FV level (24, 25), massive postpartum hemorrhage unexpectedly occurred in our female patient. Prophylactic plasma transfusion should be prepared before invasive procedures (26).

Due to the coagulation *F5* gene (1q23) and its low prevalence, only a limited number of mutations have been documented. Almost all mutations identified to date are unique and specific to each family. More than 200 genetic defects spanning the entire *F5* gene have been described. Most *F5* mutations are unique, occurring in only one patient or a few patients and family members (27). Our mutation analysis revealed multiple conserved mutation sites in the family included in our study. This study is the first to report the clinical impact of mutations in exon 16 (Met1736Val) and many mutations found in exon 13.

Initially, eight mutation sites in the patient were detected: exon 3 (Asp68His), exon 8 (Met385Thr), exon 13 (Asn789Thr), exon 13 (Lys830Arg), exon 13 (His837Arg), exon 13 (Lys897Glu), exon 16 (Met1736Val), and exon 25 (Asp2194Gly) (Table 1). Only exon 16 expressed the homozygote genotype. Other gene mutations were heterozygous. Figure 1B shows the pattern of each gene mutation sequentially. Meanwhile, the International Society on Thrombosis and Haemostasis website was searched for the genotypes of our patient with FV mutation through exon 3 (Asp68His), as this was the only well-reported mutation site (17, 28, 29) and other mutation sites were only newly discovered. The previous studies have helped us better understand FV deficiencies by documenting associated mechanisms (17, 30). For example, exon 3 (Asp68His) had been discovered, but the other seven sites have not been reported previously. Also, exon 25 (Asp2194 Gly) in recombinant FV molecules can lower the FV level (30–32). Our initial results suggest that exon 3 (Asp68His) or exon 25 (Asp2194 Gly) contribute to FV deficiency in this patient.

By comparing our patient with FV deficiencies and his family, only exon 16 (Met1736Val) was homozygous. His sister exhibited the same gene expression. Therefore, exon 16 (Met1736Val) mutation needs further evaluation to understand its contribution to FV deficiency. The previous studies regarding the genotypes of patients with FV deficiency were reviewed (16). It was reported that moderately decreased secretion rates were detected for the p.Met385Thr and p.His1299Arg FV proteins because of the quantitative defect of the p.Asp2194Gly substitution caused by the four missense variants constituting the haplotype (i.e., p.Met385Thr, p.His1299Arg, p.Met1736Val, and p.Asp2194Gly) (30). However, to our knowledge, no studies have reported about the FV deficiency related to the isolated homozygous Met1736Val; therefore, further investigation is needed.

In 2008, Liu et al. *first* identified the Asp68His mutation in Taiwan in two unrelated FV-deficient patients with compound heterozygous patterns (17). Furthermore, Cao et al. reported a homozygous pattern in a Chinese patient (28) and Huang et al. discussed a patient with a heterozygous mutation (29). Using *in vitro* recombinant mutant FV protein expression, Liu et al. demonstrated that Asp68His causes impaired secretion and ineffective FV protein translocation (17). Previously, the Asp68His mutation was reported only in a Chinese patient with FV deficiency. It is worth noting that including the two cases reported in this study (one patient from Chang Gung Memorial Hospital, Linko branch; data not shown here), four patients with heterozygous Asp68His mutation have been reported in different families. Further studies are needed to assess how the Asp68His missense mutation influences FV deficiency in Taiwanese individuals.

There remain four genotype patterns in this family, which could not be found in the genotypes of patients with FV deficiency. However, one kind of genotype, including three missense mutations (Met385Thr, Met1736Val, and Asp2194Gly), is associated with a low FV activity level (30, 31, 33, 34). Based on the results identified in the R2 haplotype allele, a low synthesis rate and impaired secretion were observed when these mutations appeared (30). Meanwhile, the Asp2194Gly mutation appears to be the major determinant of gene expression. The FV activity levels of our patients were similar to those of patients with the Asp2194Gly mutation, indicating that the phenotype influences FV activity. Other gene mutation types could be considered polymorphisms (30).

Still, many missense mutations correspond with FV deficiency. Patients with a homozygous mutation pattern have marked FV deficiency. However, the same presentation or gene mutation was not seen in our study, indicating the need to build a normal population genotype of FV in the Taiwanese population to understand gene function better. After analyzing the SNV of the normal FV population, our results indicated the uniqueness of the *F5* mutation sites that were obtained. The patient's parents also showed important heterozygous mutations in exon 3 and exon 8 but normal blood results. The difference found between the patient and his parents was that the patient's mutation was entirely inherited from his parents' mutations, indicating that the combination of mutations triggered coagulation failure. In the Keelung non-FV-deficient population, the polymorphism of Met1736Val was dominant (42.3%) but was not found in other databases. Based on a previous study, the Met1736Val mutation is associated with APC resistance, which is associated with the risk of a thromboembolic event (35). Thus, for a population with the *F5* Met1736Val mutation, such as the Keelung population, a critical issue is the prevalence rate of the risk of thromboembolism.

The prevalence of FV disease is known to be widely distinct among different populations. Also, *F5* SNVs in the Taiwanese population have not been analyzed previously. By analyzing the sequence at the *F5* locus, we found 55 SNVs from the Whole–Genome Research Core Laboratory of Human Diseases, Chang Gung Memorial Hospital, as shown in Table 3. Some non-synonymous mutations are highly conserved in the Taiwanese population, such as Arg513Lys/A2, Pro1404Ser/B, Leu1397Phe/B, Lys925Glu/B, His865Arg/B, Lys858Arg/B, and Met1764Val/A3 (R513K/A2, P1404S/B, L1397F/B, K925E/B, H865R/B, K858R/B, and M1764V/A3). Some SNVs have been recorded in the SNV database, such as rs118203905 (R334G, Arg334Gly/A1) and rs6025 (Q534R, Gln534Arg/A2), as genetic variants of the *F5* gene in Hong Kong (36). Through these two datasets, the distribution of *F5* genetic mutations is different and leads to the variable incidence of FV deficiency or FV Leiden disease.

The pathogenesis of factor V deficiency resulting from the homozygous Met1736Val mutation remains unclear. Based on the structure of the active FV obtained from the Protein Data Bank, the Met1736Val mutation site is not located in the interaction site. Moreover, the Met1736Val mutation does not influence protein synthesis (30). Further functional experiments to understand the impact of homozygous Met1736Val mutation on *F5* gene are needed. Some hypotheses are proposed to explain this result. Based on the role of FV in thrombosis, not only the homozygous Met1736Val mutation but also the seven coexisting heterozygous mutations could be caused by an abnormal interaction with FXa. First, mutated FVa cannot bind to FXa, or the weak interaction of FVa and FXa complex cannot further attach to prothrombin. However, there are no reports of identical residues for FXa binding. The current evidence shows that residues 493–506 and 311–325 in the A2 domain of FVa are important for FXa binding (37). The carboxyl-terminal Asp683–Arg709 domain of the heavy chain is essential (38) for the interaction of FVa with factor Xa and prothrombin. Therefore, in the future, functional studies can be conducted to determine whether Met1736 is crucial for either FXa or FXa and prothrombin interactions.

Finally, two cases of FV deficiency were reported, and the *F5* gene was analyzed. Multiple sites of mutations were demonstrated in the *F5* gene in one family. The mutation Asp68His was first identified as a compound heterozygous mutation in FV-deficient patients, and the homozygous pattern was previously reported in a Chinese patient (15). The patient's father carried one copy of the mutation of Asp68His, but his FV activity level was 58% (normal range: 50–150%). Other mutations contributed by the patient's mother might have led to the coagulation defect. In our study, the effect of homozygous Met1736Val mutation was associated with FV deficiency in the bleeding event rather than in the thromboembolic event mentioned before. The incidence of homozygous Met1736Val mutation is also higher in Keelung city compared with other Taiwan areas. Thus, a retrospective study is warranted to understand the influence of this mutation on the risk of thromboembolism or bleeding.

## Conclusions

New *F5* gene mutations, including homozygous Met1736Val and seven heterozygous mutations, were discovered in this study and are associated with moderate FV deficiency. The Val1736 mutation changes factor 5 protein structure and leads to coagulation disorders. Screening of the homozygote Met1736Val and the co-inheritance of the Asp68His *F5* gene is advised for patients with FV deficiency.

## Data Availability Statement

The data presented in the study are deposited in the zenodo repository, accession number: 10.5281/zenodo.6477462.

## Ethics Statement

The studies involving human participants were reviewed and approved by Chang Gung Memorial Hospital IRB (Nos. 200903793B0, 201102005A3D001, 201102005A3C102, and 201102005A3C103). The patients/participants provided their written informed consent to participate in this study.

## Author Contributions

Y-SC analyzed the data, generated the figures/tables, and wrote the manuscript. Y-CL provided the FV-deficient cases. C-NY and Y-JC performed the protein modeling and discussed the impact of gene mutation. J-SH and K-YY helped with the study design. C-YH helped develop possible pathogenesis and edited the manuscript. K-YY helped with the study design and article writing and was a significant contributor to this study. All authors read and approved the final manuscript.

## Funding

This study was supported by grants (CMRPG250251, CMRPG290061, and CMRPG2A0481) from the Chung Gang Memorial Hospital, including grants for gene testing and for results for interpretation and publication.

## Conflict of Interest

The authors declare that the research was conducted in the absence of any commercial or financial relationships that could be construed as a potential conflict of interest.

## Publisher's Note

All claims expressed in this article are solely those of the authors and do not necessarily represent those of their affiliated organizations, or those of the publisher, the editors and the reviewers. Any product that may be evaluated in this article, or claim that may be made by its manufacturer, is not guaranteed or endorsed by the publisher.

## Abbreviations

APC: activated protein C
APTT: activated partial thromboplastin time
F: factor
FV: Factor V
FX: factor X
PCR: polymerase chain reaction
PT: prothrombin time
SNV: single-nucleotide variant.
