# Supplementary material for: A Novel Phenotype of the Factor 5 Gene Mutation (Homozygote Met1736Val and Heterozygote Asp68His) Is Associated With Moderate Factor V Deficiency
Source: Front Med (Lausanne). 2022 Jun 9;9:870269. doi: 10.3389/fmed.2022.870269 (PMC9219604; doi:10.3389/fmed.2022.870269)

Supplement 1: The distribution of Factor V promoter polymorphism in Keelung non-FV deficiency population

|  |  |  | type | | | | |
| --- | --- | --- | --- | --- | --- | --- | --- |
|  |  |  | 1 | 2 | 3 | 4 | 5 |
| Promoter -1559 | -g | 111/111 | 111 | | | | |
| Promoter -1506 | t →homozygote c | 111/111 | 111 | | | | |
| Promoter -1487 | t →homozygote c | 111/111 | 111 | | | | |
| Promoter - 790 | -c | 111/111 | 111 | | | | |
| Promoter -319 | a →homozygote c | 1/111 | 0 | 0 | 1 | 0 | 0 |
| Promoter -281 | g →homozygote a | 74/111 | 72 | 0 | 1 | 0 | 1 |
| Promoter -95 | t →homozygote c | 109/111 | 72 | 36 | 1 | 0 | 0 |


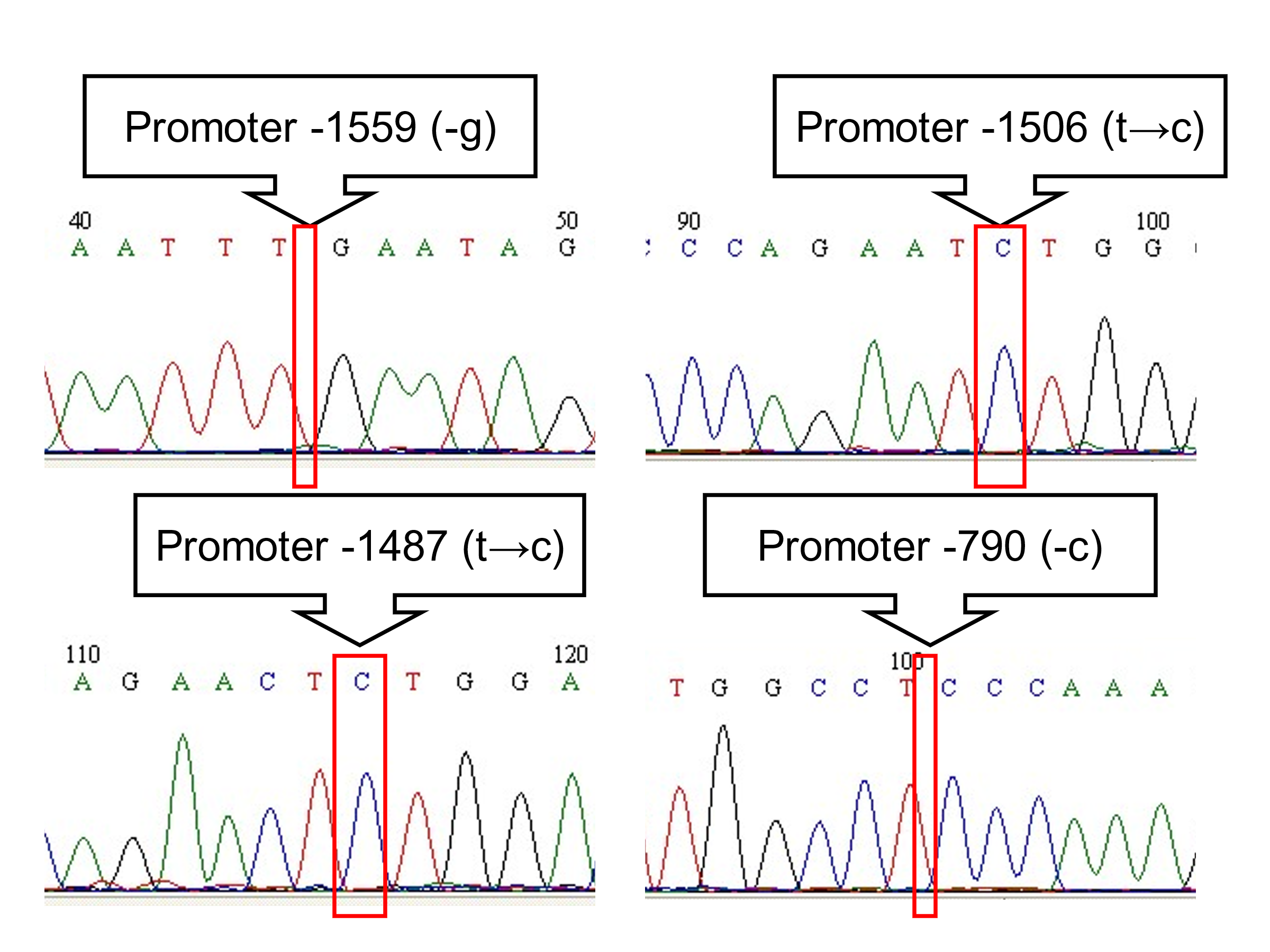

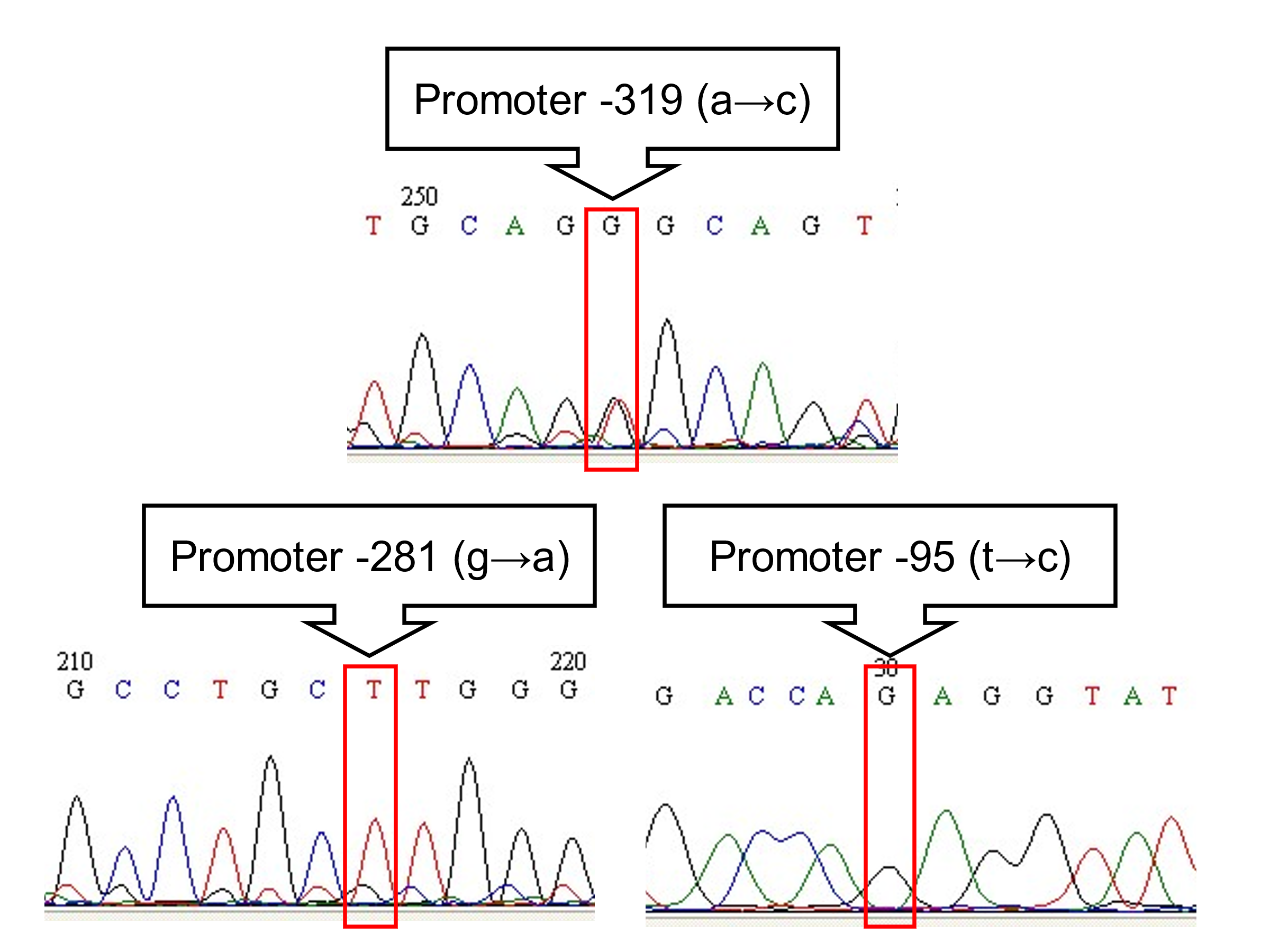

Supplement: Supplementary file 1 [file Data_Sheet_1.docx]
